# Barriers to adequate follow-up during adjuvant therapy may be important factors in the worse outcome for Black women after breast cancer treatment

**DOI:** 10.1186/1477-7819-6-26

**Published:** 2008-02-25

**Authors:** Steve H Kim, Jeanne Ferrante, Bok Ran Won, Meera Hameed

**Affiliations:** 1 Department of Surgery, Geisinger Wyoming Valley Medical Center, Wilkes Barre, PA, 18711, USA; 2 Department of Family Medicine, University of Medicine and Dentistry of New Jersey/New Jersey Medical School, Newark, NJ, 07103, USA; 3 Department of Radiology, University of Medicine and Dentistry of New Jersey/New Jersey Medical School, Newark, NJ, 07103, USA; 4 Department of Pathology, University of Medicine and Dentistry of New Jersey/New Jersey Medical School, Newark, NJ, 07103, USA

## Abstract

**Introduction:**

Black women appear to have worse outcome after diagnosis and treatment of breast cancer. It is still unclear if this is because Black race is more often associated with known negative prognostic indicators or if it is an independent prognostic factor. To study this, we analyzed a patient cohort from an urban university medical center where these women made up the majority of the patient population.

**Methods:**

We used retrospective analysis of a prospectively collected database of breast cancer patients seen from May 1999 to June 2006. Time to recurrence and survival were analyzed using the Kaplan-Meier method, with statistical analysis by chi-square, log rank testing, and the Cox regression model.

**Results:**

265 female patients were diagnosed with breast cancer during the time period. Fifty patients (19%) had pure DCIS and 215 patients (81%) had invasive disease. Racial and ethnic composition of the entire cohort was as follows: Black (N = 150, 56.6%), Hispanic (N = 83, 31.3%), Caucasian (N = 26, 9.8%), Asian (N = 4, 1.5%), and Arabic (N = 2, 0.8%). For patients with invasive disease, independent predictors of poor disease-free survival included tumor size, node-positivity, incompletion of adjuvant therapy, and Black race. Tumor size, node-positivity, and Black race were independently associated with disease-specific overall survival.

**Conclusion:**

Worse outcome among Black women appears to be independent of the usual predictors of survival. Further investigation is necessary to identify the cause of this survival disparity. Barriers to completion of standard post-operative treatment regimens may be especially important in this regard.

## Introduction

"Racial, ethnic, and socioeconomic disparities are national problems that affect health care at all points in the process." This declaration came from the first National Healthcare Disparities Report released by the U.S. Department of Health and Human Services in 2003, and was supported by literature from a wide variety of medical specialties. [1] More specifically, African-American or Black women have had historically worse outcome after treatment for breast cancer when compared to their non-Black counterparts. [2-8] A number of putative, probably inter-related factors have been implicated to explain this discrepancy including genetic background [9-12], diet and body habitus [13-16], cultural attitudes toward cancer and medicine in general [17-21], poverty and limited access to healthcare [22-26], late stage at presentation[23,27,28], as well as a various intrinsic biological properties of the primary tumor. [29-36] To try and further elucidate this issue, we examined the outcomes for this disease within our institution. The University Hospital in Newark, New Jersey is a major source of healthcare for low income and medico-economically underserved patients, the great majority of whom are Black or Hispanic. Given the relative uniformity in socio-economic status of the patient population, potentially valuable insight might be gained by examining the comparative outcome of Black women in this cohort study.

## Methods

University Hospital is a tertiary care medical center that is New Jersey's only public hospital and receives the largest share of charity care funding of any facility in the state. [37] Using retrospective cohort analysis of a prospectively collected database, we examined the outcomes of treatment for operable breast cancer at this institution from May 1999 through June 2006. Patients not having surgical resection were excluded. Race and ethnicity were classified at initial patient registration via self-identification. The following racial categories were utilized as per the Federal Office of Management and Budget guidelines: American Indian or Alaskan Native, Asian, Black or African American, Native Hawaiian or Other Pacific Islander, and White. [38] Patients were also ethnically classified as Hispanic or non-Hispanic[38]; those women who were of Black race but Hispanic ethnicity were categorized as Black for analytic purposes (Table 1). Other patient and tumor characteristics were collected prospectively from patient charts and pathology reports and are summarized in Tables 1, 2, 3, and 4. Follow up status was obtained via physician visit notes and patient interview. Recurrences were confirmed pathologically whenever indicated; otherwise, a highly suggestive imaging study leading to further treatment was used as documentation. Survival was analyzed via the method of Kaplan-Meier. [39] Death from disease was the main endpoint (disease-specific survival). Statistical significance was determined by chi-square analysis when examining differences in patient and tumor characteristics between races, log rank testing for survival analysis, and the Cox regression model for multivariate outcome analysis. The study was performed with IRB approval.

**Table 1:** Racial/ethnic composition.

Identifier	N (%)
Black	150 (56.6%)
Hispanic (non-Black)	83 (31.3%)
Caucasian (non-Hispanic)	26 (9.8%)
Asian	4 (1.5%)
Middle-Eastern (Arabic)	2 (0.8%)

**Table 2:** Patient factors examined by race.

	Black	Others	p
Mean (Median) Age (yrs), all patients	54.2 (53.4)	53.6 (51.1)	.70
Mean (Median) Age (yrs), invasive disease	54.0 (53.4)	52.9 (51.2)	.55
% of patients ≤ 50 Y	41% (62/150)	50% (57/115)	.18
			
Mean (Median) BMI, all patients	31.6 (30.7)	29.2 (27.9)	<.05
Mean (Median) BMI, patients ≤ 45 Y	30.6 (30.6)	29.7 (29.2)	.60
Mean (Median) BMI, patients > 45 Y	32.0 (30.8)	28.9 (27.5)	<.01
			
Medical Comorbidities (≥ 1)	82/150 (55%)	48/115 (42%)	<.05
Medical Comorbidities (≥ 2)	34/150 (23%)	11/115 (10%)	<.01
Hypertension	69/150 (46%)	35/115 (30%)	<.05
Diabetes	15/150 (10%)	15/115 (13%)	.44
Cardiac disease or PVD	18/150 (12%)	6/115 (5%)	.06
Renal insufficiency	4/150 (3%)	1/115 (1%)	.29
Hepatitis and/or cirrhosis	6/150 (4%)	1/115 (1%)	.12
Reactive airway disease/COPD	15/150 (10%)	6/115 (5%)	.15
			
Contralateral Breast Cancer, all	8/150 (5%)	7/115 (6%)	.79
Contralateral Breast Cancer, synchronous	1/150 (1%)	5/115 (4%)	<.05
Contralateral Breast Cancer, metachronous	7/150 (5%)	2/115 (2%)	.19
			
Family History of Breast Cancer, any	41/150 (27%)	25/115 (22%)	.21
Family History of Breast Cancer, 1° relatives	15/41 (37%)	10/25 (40%)	.92
			
Health insurance, Uninsured	52/150 (35%)	66/115 (57%)	<.001*
Health insurance, Medicaid	38/150 (25%)	19/115 (17%)	
Health insurance, Medicare	46/150 (31%)	15/115 (13%)	<.05^#^
Health insurance, Private	14/150 (9%)	15/115 (13%)	
			
Screening Detected, all patients	55/150 (37%)	48/115 (42%)	.40
Screening Detected, invasive disease only	36/124 (29%)	31/91 (34%)	.43

* Comparison of fraction of uninsured patients in each group.

^# ^Comparison of fraction of patients who were uninsured or had Medicaid vs. those who had Medicare and/or private insurance in each group.

**Table 3:** Patterns of Breast Surgery, Axillary Staging, and Adjuvant Therapy.

Disease	Treatment	Black	Other
DCIS	BCT	15/26 (58%)	14/24 (58%)
	MAST	9/26 (34%)	7/24 (29%)
	MAST-R	2/26 (8%)	3/24 (13%)
			
Invasive	BCT	57/124 (46%)	41/91 (45%)
	MAST	54/124 (44%)	33/91 (36%)
	MAST-R	13/124 (10%)	17/91 (19%)
			
Invasive	SLNB	51/124 (41%)	34/91 (38%)
	SLNB + ALND	30/124 (24%)	31/91 (34%)
	ALND	39/124 (32%)	22/91 (24%)
	None	4/124 (3%)	4/91 (4%)
			
Invasive	CT	91/124 (73%)	65/91 (71%)
	RT	76/124 (61%)	54/91 (59%)
	HT	67/124 (54%)	53/91 (58%)
	NC	19/124 (15%)	3/91 (3%) *

Key: BCT – breast conservation therapy, MAST – mastectomy, MAST-R – mastectomy with reconstruction, SLNB – sentinel lymph node biopsy only, SLNB + ALND – sentinel lymph node biopsy and axillary node dissection, ALND – axillary node dissection only, CT – Chemotherapy offered and accepted, RT – radiation therapy offered and accepted, HT – Hormonal therapy offered and accepted, NC – noncompliance with offered adjuvant therapy.

* The difference in the incidence of noncompliance was statistically significant, p < .01.

**Table 4:** Pathologic factors examined by race.

	Black	Other	p
Mean primary tumor size (median)	3.0 cm (2.4)	3.0 cm (2.5)	.92
% of tumors which were multifocal	38% (40/106)	32% (25/77)	.69
% of tumors which were high grade	44% (46/104)	36% (26/73)	.25
% of tumors which were ER-negative	42% (50/118)	34% (30/87)	.25
% of tumors which were Her2-positive	25% (28/112)	25% (21/84)	1.00
% node-positive disease	51% (63/124)	47% (43/91)	.61
Mean (median) number of harvested nodes in patients undergoing ALND	21.3 (20)	21.8 (21)	.76
Mean (median) number of involved nodes in node-positive patients	5.1 (2)	4.0 (2.5)	.39

## Results

### Patient factors

Between May 1999 and June 2006, 265 women underwent operative therapy for breast cancer at University Hospital in Newark. Of these, 215 patients had invasive disease (81%) and 50 patients (19%) had pure ductal-carcinoma-in-situ (DCIS). Racial/ethnic composition of the cohort is listed in Table 1. Black females made up the majority of our cohort (57%, N = 150), and all subsequent analyses grouped the remaining non-Black racial/ethnic categories together (N = 115) so as to be able to perform pair-wise comparison of factors and outcomes. The mean age of the Black patients was 54.2 years as compared to 53.6 years for the rest. Among the subset with invasive cancers (N = 215), mean age was 54.0 years for Black patients and 52.9 years for others. Patients under 50 years of age made up 41% of the former cohort (N = 62/150) and 50% of the latter (N = 57/115); among the patients with invasive tumors, these fractions were 41% (N = 51/124) vs. 48% (N = 44/91), respectively. None of these differences were statistically significant (Table 2).

Although definitive data on socio-economic status was not available, we felt that health insurance status might act as an adequate surrogate for this factor. At the time of initial treatment, 45% (N = 118) of the entire cohort was uninsured ("charity care"), 21% (N = 57) had Medicaid or were covered by a Medicaid HMO, 23% (N = 61) had Medicare, and 11% (N = 29) had private insurance. Black patients were significantly less likely to have no insurance (charity care) than others (35% vs 57 %, respectively, p < .001). If Medicaid and charity care are categorized together and compared to patients insured by Medicare or private insurance, Black patients were still more likely than the others to be in the latter group (40% vs 26%, respectively, p < .05). Despite these notable race-based discrepancies in level of insurance coverage, there was no significant difference between the two groups in terms of percentage of cancers found on screening mammography vs. those discovered clinically by the patient or physician (Table 2, p = .41).

Obesity has been linked to both a higher risk of breast cancer diagnosis [40-42] and disease-specific mortality. [43-48] Concordantly, the median BMI of our entire cohort was 30 (range 16.1 – 68.8), a number that falls into the "obese" category (Table 2). [49] Black females however, were significantly more obese than those of other racial/ethnic groups (median BMI 31.6 vs. 29.2, p < .05). Subset analysis, however, demonstrated that there was no race-based difference in BMI in women younger than 45 years (30.6 for Blacks, 29.7 for others). The higher incidence of obesity was mainly limited to Black women > 45 years of age (median BMI 32.0 vs 28.9 for the others, p < .01).

Overall, Black women also had a significantly higher incidence (55% vs. 42%, p < .05) of a history of at least one of the following medical comorbidities: 1) hypertension, 2) diabetes, 3) cardiac or peripheral vascular disease, 4) renal insufficiency, 5) hepatitis or cirrhosis, and/or 6) reactive airway disease or chronic obstructive pulmonary disease (COPD). More specifically, they were at higher risk for hypertension (46% vs. 30%, p < .05) and borderline higher risk for significant cardiac or peripheral vascular disease (12% vs. 5%, p = .06). Black women were also more likely to be affected by two or more concomitant comorbidities (23% vs. 10%, p < .01).

A history of contralateral breast cancer was found in 15 patients (6%). Of these, 9 were metachronous and 6 were synchronous. There was no significant difference between the patient subsets in the incidence of bilateral disease – 8/150 (5%) for Black women and 7/115 (6%) for others. However, the risk of *synchronous *bilateral breast cancer was higher in the non-Black patient group (p < .05, Table 2). Of the patients who had knowledge of their family history (N = 260, 98%), 25% (N = 66/260) claimed at least one family member with a diagnosis of breast cancer. There was no statistically significant difference in the fraction of Black women with a positive family history of disease (27%, N = 41/150) as compared to women of other races (22%, N = 25/115). Of the 66 women who did give a positive family history, 38% (N = 25) had at least one first-degree relative affected. Again, there was no significant racial difference in the number of women who had at least one affected first-degree relative vs. those in whom the affected relative was more distant: N = 15/41 (37%) for Black patients and N = 10/25 (40%) for others (Table 2).

### Diagnosis and treatment

Among the patients with invasive disease (N = 215), only 67 (31%) had their tumors initially detected with screening mammography. The great majority of the patients presented with a self-discovered mass as the initial complaint (N = 135, 63%). The rest had either a mass detected on physical exam (N = 10, 5%) or persistent mastalgia as the main complaint (N = 3, 1%). No significant racial discrepancy was found in the incidence of screening-detected cancers: 29% (36/124) for Black women compared to 34% (31/91) for others. The interval from date of initial abnormal mammogram or breast physical exam to date of diagnostic biopsy was not specifically examined in this study. However, a previous report has demonstrated no race-based differences in this time interval at our institution. [50] Excluding those patients who were diagnosed at outside hospitals (N = 34) and those who underwent neoadjuvant chemotherapy (N = 34), the mean interval from date of pathologic diagnosis to date of initial surgical treatment was 25 days (median 22 days) for the entire cohort. This interval was not statistically changed based on race (median of 23 days for Black patients vs. 22 days for the rest) or insurance status (charity care vs. Medicaid vs. Medicare/private insurance).

Of the 50 patients with pure DCIS, 29 (58%) had breast-conserving surgery, while 21 (42%) had mastectomy (5 of the 21 had immediate reconstruction at the time of resection). Breast surgery in patients with invasive disease (N = 215) was as follows: local excision (N = 98, 46%), mastectomy (N = 89, 41%), and mastectomy with immediate reconstruction (N = 28, 13%). In 85 (40%) of these patients, axillary staging was accomplished with sentinel lymph node biopsy (SLNB) only. In the remainder of the patients with invasive breast cancer, 61 (28%) had SLNB and axillary lymphadenectomy (ALND), 61 (28%) had ALND only, and 8 (4%) did not undergo an axillary staging procedure. Patterns of surgery are summarized in Table 3. No statistically significant difference was noted in use of breast conservation surgery based on patient race.

Indications for neoadjuvant chemotherapy were either locally advanced or unresectable disease at presentation or a resectable large primary tumor that precluded breast conservation in a patient who was strongly adverse to mastectomy. Neoadjuvant chemotherapy was given to 18% of Black females (22/124) and 13% of women of other ethnicities (12/91). This difference was not statistically significant.

### Histopathology

Standard prognostic factors were examined and compared for the two groups (Table 4). Mean primary tumor size was 3.0 cm and was the same for both subsets (median tumor size was 2.4 cm for Black women and 2.5 cm for others). For patients in whom this information was specified on the pathology report (N = 183), the incidence of multifocal disease (defined as discontinuous foci of either DCIS or invasive disease) was not significantly different based on race, being found in 38% (40/106) of Black patients and 32% (25/77) of the others. There was a similarly higher fraction of Black patients with high-grade tumors (44 vs. 36%) and with tumors that were estrogen-receptor (ER) negative (42% vs. 34%), however, these differences were not statistically significant. Her2 expression was noted in 25% of both patient cohorts.

Lymph node status was also similar for Black women and other patients. The nodal staging was technically adequate; the mean and median number of total harvested axillary nodes was approximately 21 in both groups (Table 4). Node-positive disease was found in 51% of the Black females and 47% of the others (p = .61). The mean number of positive nodes in Black women was 5 compared to 4 for others (median 2 vs. 2.5, respectively, p = .39). In summary, standard primary tumor and nodal factors were statistically similar between Black and non-Black patient subsets.

### Follow-up and outcomes

Survival and outcome analysis was limited to patients with invasive disease (N = 215). Mean follow-up time was 2.7 years. The fraction of patients receiving adjuvant therapy was as follows: hormonal treatment was given to 120 patients (56%), systemic chemotherapy (either pre-operatively or post-operatively) to 156 patients (73%), and radiation therapy to 130 patients (60%). Response to neoadjuvant therapy was defined as a measurable decrease in T and/or N stage after chemotherapy, as determined by comparison of the pre-operative physical exam and radiological studies with the final pathology report. For instance, a decrease in FDG-uptake on PET scan was not considered a response unless it was accompanied by a definite decrease in size and/or significant histopathologic necrosis of the final specimen. By this measure, there was a notable difference between the two cohorts, i.e., only 9/22 (41%) Black women responded, whereas 8/12 (67%) women of other races showed a dramatic response. These differences did not reach statistical significance (p = .15), likely due to the small numbers involved. Compliance with post-operative adjuvant therapy was defined as completion of recommended treatment in a timely manner (morbidity-related delays due to wound infections or neutropenia were not counted as non-compliant). With this definition, we documented non-compliance in only 3/91 (3%) non-Black females. However, 19/124 (15%) Black females either refused or failed to complete standard post-operative adjuvant therapy regimens, a highly significant disparity (Table 3, p < .01). As might be expected, failure to complete adjuvant therapy was significantly related to the risk of locoregional recurrence – 6/22 patients (27%) vs. 7/193 patients (4%), p < .001. Concordantly, Black women were significantly more likely to have a locoregional recurrence within the time frame of our follow-up than those of other races (10% vs. 1%, p < .01, Table 5).

**Table 5:** Locoregional and distant recurrence data between racial groups.

Recurrence type	Black	Other	p
None, N (%)	101/124 (81%)	85/91 (93%)	.01
Isolated locoregional	6/124 (5%)	1/91 (1%)	.13
Any locoregional	12/124 (10%)	1/91 (1%)	<.01
Distant	18/124 (15%)	5/91 (5%)	.03

**Figure 1:**
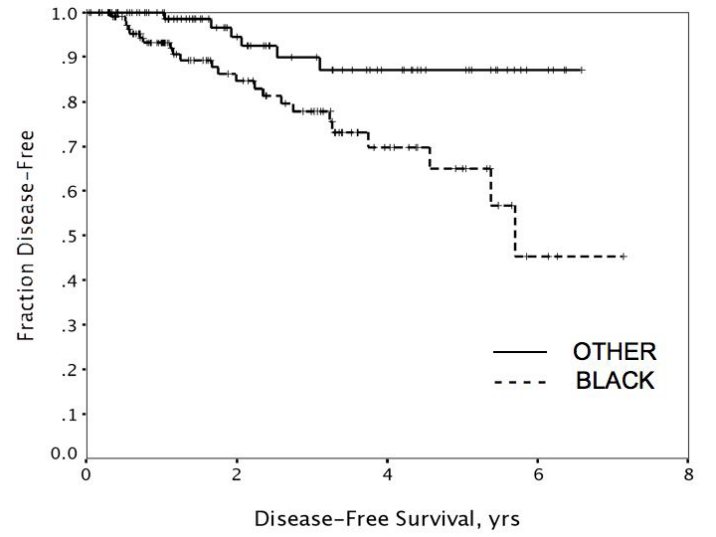
Disease-free survival was significantly worse in Black women compared to those of other races (p < .01).

**Figure 2:**
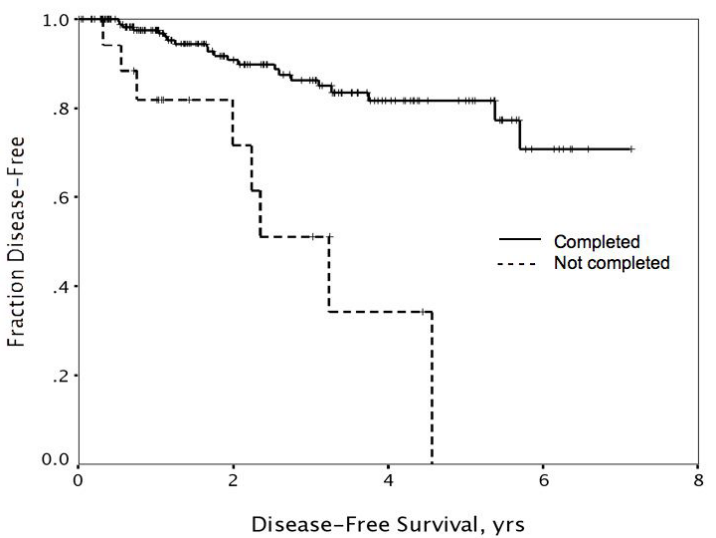
Refusal or failure to complete adjuvant therapy regimens was associated with significantly worse rates of disease-free survival (p < .001).

**Figure 3:**
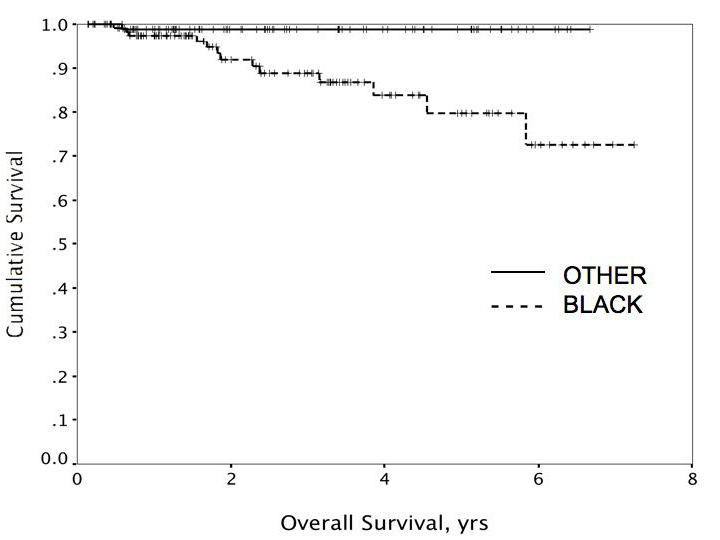
Black race was a significant predictor of disease-specific survival on both univariate (p < .01) and multivariate analyses.

Factors *not *predictive of either disease-free or overall survival by univariate analysis included presence of two or more comorbidities, body mass index (BMI) > or ≤ 30, lack of insurance, tumor grade, and hormone receptor status. Univariate factors predicting worse disease-free survival (DFS) included increasing tumor size, node-positivity, Black vs. other race (Figure 1), and non-compliance with adjuvant treatment (Figure 2). When subjected to multivariate analysis, all remained independent predictors of disease-free survival (Table 6). Univariate predictors of overall disease-specific survival included tumor size, node-positivity, and Black vs. other race (Figure 3). Non-compliance was not predictive when disease-specific death was the endpoint (p = .13 and p = .64, respectively). In multivariate analysis, primary tumor size, node-positivity, and Black race were all independent predictors of overall breast cancer specific survival to varying degrees (Table 6).

**Table 6:** Multivariate analysis of factors associated with disease-free survival and disease-specific overall survival.

Factor	DFS (p)	HR (95% CI)	OS (p)	HR (95% CI)
Tumor size	.03	1.1 (1.0 – 1.2)	<.01	1.2 (1.1 – 1.3)
Node-positive disease	.04	1.5 (1.0 – 2.3)	.02	2.2 (1.1 – 4.5)
Black race	.01	0.38 (0.15 – 0.95)	.03	0.12 (0.02 – 1.0)
Compliance with adjuvant therapy	<.01	4.1 (1.8 – 9.7)	NS	

## Discussion

Race-based analysis of healthcare outcomes has long been a source of controversy. Complicating the matter is the impossibility of establishing absolutely clear ethnic, cultural, or genetic boundaries that allow definitive categorization of patients based on the concept of "race." Some authors have considered the present boundaries arbitrary at best, and even fraught with potential moral ambiguity, if one accepts the concept of race as a "societal construct."[51,52] Others, however, have considered these categorizations informative, arguing that racial differences in disease course and response to therapy empirically exist and may have a genetic basis. [53] Clearly, the main issue is the relationship of "race" to other known factors of poor prognosis, that is, are they independent or simply associative? In this study, we attempt to examine this issue within our database of breast cancer patients. We acknowledge some limitations to this analysis. Our patient population was largely Black and Hispanic; Caucasians and Asians made up only about 10% of the cohort. Thus, when we compare Black patients to non-Black patients, this is largely a comparison of the former group to non-Black Hispanics. Secondly, we realize that our follow up time is relatively short for a study of breast cancer outcomes. Despite these caveats, however, some interesting results are evident.

Within the context of our largely urban, poor patient cohort, Black women tended to have better financial healthcare coverage than those of other races. Over half of them had some form of insurance compared to only about a third of the others. Nevertheless, most patients were uninsured or had Medicaid, and it was not surprising that a minority of women in both groups had screening detected cancers (29% of Black women and 34% of others, p = .43, Table 2). Although the median age of the two groups was similar, Black women were more likely to have one or more medical comorbidities such as hypertension or diabetes and also tended to be more obese (albeit, the latter finding was mainly limited to post-menopausal women, Table 2). Body mass index and the presence of comorbidities were not significant predictors of disease-free or overall survival, however.

There were no race-based differences in time intervals to diagnosis and start of treatment. In a previous study from our group that included patients from the present cohort, we examined the time interval between date of initial suspicious mammogram and/or physical exam and final pathologic diagnosis (the diagnostic interval) and found this to be statistically equivalent among women of different races. [50] In the present study, we further demonstrated no racial difference in time to definitive surgery after the pathologic diagnosis of breast cancer had been established. The nature of the subsequent surgical treatment and pathology results was also statistically similar between racial groups (Tables 3 and 4).

Recent data has suggested that minority women (Blacks especially, but Hispanics as well) were significantly less likely to be offered appropriate postoperative therapy than Caucasians. [54] In our cohort, we found that adjuvant radiation and chemotherapy were offered and given to a similar fraction of patients in each group (Tables 3). Other studies have noted that chemotherapy doses are often significantly lower in Black women and have implicated this factor as a source of prognostic disparity. [55] We could not address this issue, unfortunately, as information on specific doses was not recorded in our database. However, one striking feature of our patient population was the rate of noncompliance with post-operative adjuvant therapy (either outright refusal or failure to complete therapy) in Black women. This was noted in 15% of this group but only 3% of women of other races (Table 3, p < .01). This racial discrepancy in the fidelity of post-operative follow-up has been previously noted by other authors and implicated as a possible cause of outcome disparities. [25]

Black race along with expected factors such as tumor size and lymph node status were significant independent determinants of disease-free and overall survival (Table 6). Factors such as hormone receptor negativity and high grade/poor tumor differentiation did not reach statistical significance, a finding we ascribe to our relatively small data set and follow up time. Insurance status, presence of comorbidities, and body mass index were not significant predictors of outcome. Not surprisingly[25,56], lack of compliance with postoperative adjuvant therapy had significant negative impact on the chance of disease-free survival in both univariate and multivariate analyses (Table 6). Interestingly, multivariate analysis of *overall *disease-specific survival demonstrated only tumor size, nodal status, and Black race to be significant. When examining the results of the Cox regression analysis, Black race was associated with only a slightly worse prognosis based on hazard ratios (38% higher risk of disease recurrence and only 12% higher risk of death due to breast cancer). However, noncompliance with adjuvant therapy conferred a greater than 4× higher risk of disease recurrence – significantly greater than even tumor size (HR 1.1) or nodal status (HR 1.5, Table 6). Given the high correlation of Black race to noncompliance, we are therefore somewhat circumspect as to the ultimate relation between these factors and overall survival. Although noncompliance appeared to lose significance in the Cox regression analysis of overall survival, we suspect that this is mainly a function of follow up time, with eventual deaths from recurrence being inevitable.

We are uncertain as to why Black women in this study had such a high rate of failure to complete adjuvant therapy. Obviously, postoperative treatment is a difficult process that requires serious and time-intensive patient commitment. The more frequent utilization of less-than-radical surgery, although welcome, has only made this more problematic since conservative resection is frequently combined with more rigorous and demanding adjuvant treatment regimens. We can speculate on a number of reasons why Black women may be less compliant with these demands. Although the great majority of our patient cohort could easily be described as underserved, there was little question that Black patients were not over-represented in this regard, and in fact, were more likely to have some form of insurance than those of other groups (Table 2). Furthermore, there were many more English-speaking patients in this racial group, thus one could reasonably surmise that language was not a significant barrier to appropriate post-operative treatment. Other more formidable barriers may exist, however. Historical data suggests that healthcare in the Black community may be undermined by mistrust and/or lack of faith in the medical establishment, much of it stemming from revelations of the Tuskegee syphilis experiments in which untreated Black men were unknowing subjects of a natural history study by the United States Public Health Service. This attitude may be more pervasive than previously realized, especially among those of lower socioeconomic status [57-59]. Finally, although clearly important, we cannot comment on the levels of familial and social support available to patients during their treatment.

In conclusion, although we found Black race to be a predictor of poor outcome after treatment for breast cancer, it had a relatively small effect as an independent factor. Failure to follow through with postoperative adjuvant therapy was the most important factor in determining recurrence-free survival, and this factor was significantly more prevalent in our Black patient cohort. Further research should be aimed at seeing if this phenomenon is more generally observed, examining reasons why this may occur, and implementing potential solutions. In this regard, use of patient navigators may be a promising intervention. [60,61] It is also imperative that larger prospective studies continue to identify and address the socio-cultural and/or biologic factors that continue to cause racial discrepancies in cancer outcome.

## Competing interests

The author(s) declare that they have no competing interests.

## Authors' contributions

SHK conceived the project, had full access to all of the data in the study, and takes responsibility for the integrity of the data and the accuracy of the data analysis. JF participated in the design of the project. BRW and MH participated in the collection of the raw data. All authors participated in the data analysis and also read and approved the final manuscript.
